# Fusion Gene Detection Using Whole-Exome Sequencing Data in Cancer Patients

**DOI:** 10.3389/fgene.2022.820493

**Published:** 2022-02-16

**Authors:** Wenjiang Deng, Sarath Murugan, Johan Lindberg, Venkatesh Chellappa, Xia Shen, Yudi Pawitan, Trung Nghia Vu

**Affiliations:** ^1^ Department of Medical Epidemiology and Biostatistics, Karolinska Institutet, Stockholm, Sweden; ^2^ Biostatistics Group, Greater Bay Area Institute of Precision Medicine, Fudan University, Guangzhou, China; ^3^ Centre for Global Health Research, Usher Institute, University of Edinburgh, Edinburgh, United Kingdom

**Keywords:** fusion gene, acute myeloid leukemia, whole exome sequencing, prostate cancer, discordant read, split read

## Abstract

Several fusion genes are directly involved in the initiation and progression of cancers. Numerous bioinformatics tools have been developed to detect fusion events, but they are mainly based on RNA-seq data. The whole-exome sequencing (WES) represents a powerful technology that is widely used for disease-related DNA variant detection. In this study, we build a novel analysis pipeline called Fuseq-WES to detect fusion genes at DNA level based on the WES data. The same method applies also for targeted panel sequencing data. We assess the method to real datasets of acute myeloid leukemia (AML) and prostate cancer patients. The result shows that two of the main AML fusion genes discovered in RNA-seq data, PML-RARA and CBFB-MYH11, are detected in the WES data in 36 and 63% of the available samples, respectively. For the targeted deep-sequencing of prostate cancer patients, detection of the TMPRSS2-ERG fusion, which is the most frequent chimeric alteration in prostate cancer, is 91% concordant with a manually curated procedure based on four other methods. In summary, the overall results indicate that it is challenging to detect fusion genes in WES data with a standard coverage of ∼ 15–30x, where fusion candidates discovered in the RNA-seq data are often not detected in the WES data and vice versa. A subsampling study of the prostate data suggests that a coverage of at least 75x is necessary to achieve high accuracy.

## 1 Introduction

Fusion genes represent an important class of genomic alteration contributing to the tumorigenesis for both solid and hematological cancers. The hybrid genes are often produced by recurrent chromosomal rearrangements, such as translocation, deletion and insertion (De Braekeleer et al., 2011; Sonoda et al., 2018; Suo et al., 2018). In the early 1980s, the first fusion gene was discovered in patients with chronic myeloid leukemia (CML), which was caused by the translocation between chromosome 9 and 22. The fusion gene, BCR-ABL1, plays a prominent role in inducing the chronic myeloid leukemia (Chandran et al., 2019). In the last decades, a great number of fusion genes with functional impacts have been detected in different cancers. For example, the TMPRSS2-ERG, which originates from an interstitial deletion in chromosome 21, has been identified in ∼50% of prostate cancer cases (Kron et al., 2017). In the high-grade serous ovarian cancer, about 7% of patients carrying the BCAM-AKT2 fusion, which is specific and unique for this cancer type (Kannan et al., 2015; Mohajeri et al., 2013). Recent studies have revealed the importance and significance of fusion gene to serve as diagnostic marker and drug target (Bruno and Fontanini 2020). A comprehensive characterization of fusion genes can facilitate the molecular diagnosis and improve customized therapy for cancer patients.

The advent of next generation sequencing has greatly accelerated the discovery of genomic mutations underlying human diseases. In the last several years, the RNA-seq data have been widely used to detect fusion events. Multiple bioinformatics tools are developed to predict fusions from RNA-seq data, such as TopHat-Fusion (Kim and Salzberg 2011), JAFFA (Davidson et al., 2015), STAR-Fusion (Haas et al., 2017) and Fuseq (Vu et al., 2018). Generally, according to their detection strategies, these methods can be divided into two categories: 1) mapping-first method and 2) *de novo* assembly-first method (Kumar et al., 2016). In the mapping-first approach, the RNA-seq reads are first mapped to the reference genome or transcriptome. The discordantly mapped reads including spanning and split reads are then extracted to predict the fusion genes (Haas et al., 2019). For the *de novo* assembly-first approach, the reads are first assembled into longer transcripts, which are then compared with the reference sequence to identify candidate fusion events. Although these methods achieve favorable results when detecting chimeric genes, further improvements in accuracy and performance are still needed to produce more reliable estimates (Carrara et al., 2013).

Apart from RNA sequencing, the whole-exome sequencing (WES) represents another primary type of sequencing application which has been frequently utilized in cancer studies (Bao et al., 2014). The experiment of WES contains two major steps: 1) capturing the protein-coding region and 2) sequencing the reads at deep level using high-throughput sequencing platforms. The use of WES achieves big success in identifying complex mutational signatures associated with various diseases, such as breast cancer and Alzheimer’s disease (Dieci et al., 2016; Raghavan et al., 2018). Compared with the whole-genome sequencing, WES only sequence the exon region, which accounts for < 2% of the whole genome. This feature makes the whole-exome sequencing much more cost-effective and practical for large-scale usage in medical research and clinical diagnostics.

In our previous study, we have developed a method named Fuseq to identify fusion genes from RNA-seq data, which provides an accurate and fast prediction of the fusion aberration (Vu et al., 2018). In this study, we aim to evaluate the potential value of exome sequencing data for the purpose of fusion detection. We construct a novel analysis method, named Fuseq-WES, and implement it to several cancer type cohorts including acute myeloid leukemia (AML) and prostate cancer. We find that the fusion genes detected from WES data are concordant with those from RNA-seq data. For example, two of the major fusion genes, PML-RARA and CBFB-MYH11, are validated in 36 and 63% of the available samples, respectively. Detection of the well-established fusion gene TMPRSS2-ERG in prostate cancer using targeted deep DNA-sequencing data has 91% concordance manually curated calls of the fusion. A subsampling study of the prostate data suggests that a coverage of at least 75x is necessary for Fuseq-WES to achieve high accuracy.

## 2 Materials and Methods

### 2.1 Overview of Fuseq-WES Pipeline

**FIGURE 1:**
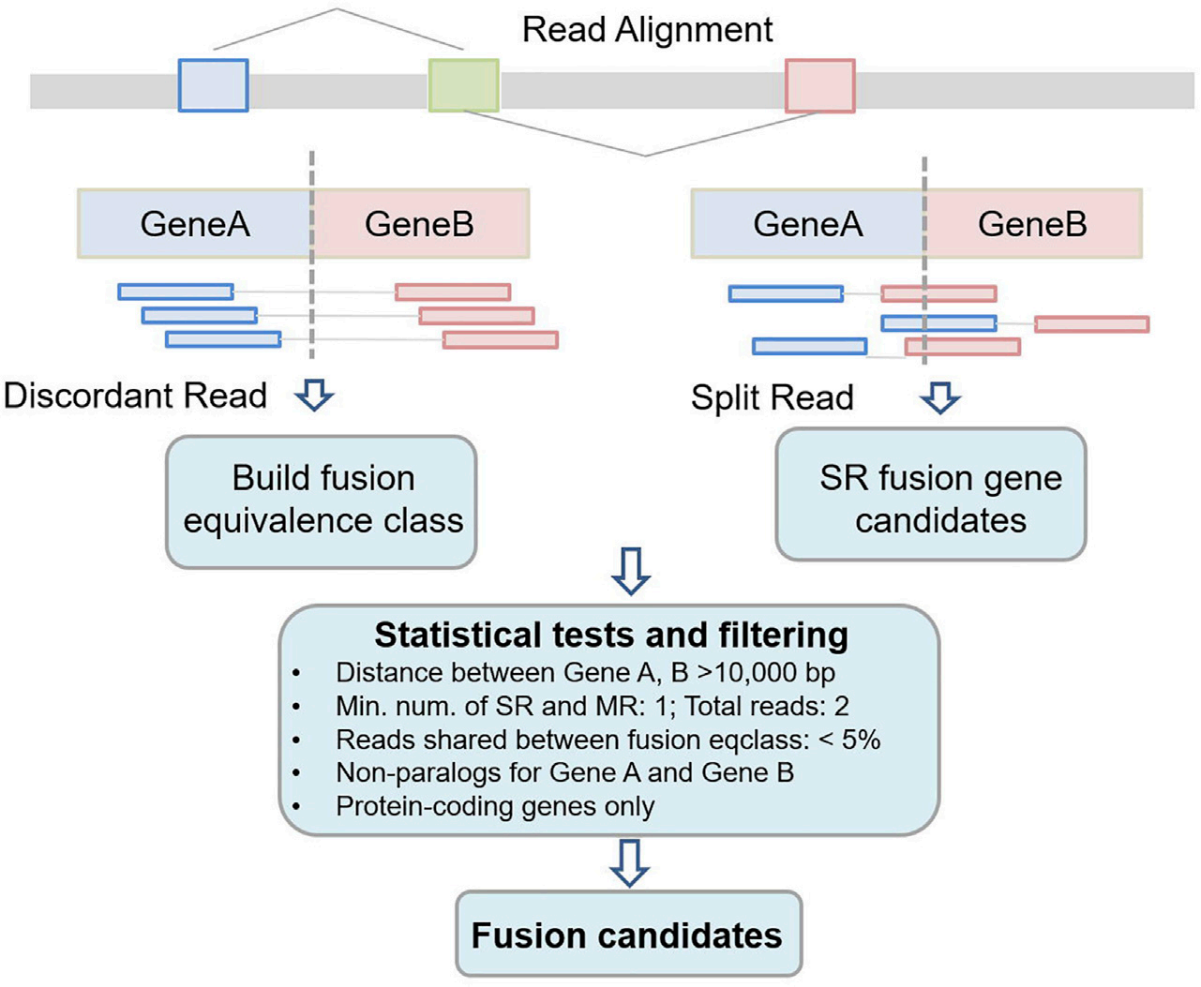
Workflow of Fuseq-WES to detect fusion genes from whole-exome sequencing data.

Figure 1 shows the workflow of Fuseq-WES method. There are four key steps in the pipeline: 1) extraction of discordant and split reads from read alignment; 2) reads annotation and build the fusion equivalence class; 3) apply various statistical tests and filters to remove false positive fusion candidates and 4) output the final fusion gene lists.

### 2.2 Extraction of Discordant and Split Reads

Reads mapping is the process to infer which region in the reference sequence that a read can originate from. In recent years, a wide range of computational tools have been developed and implemented for reads mapping, e.g., BWA, HISAT2 and Bowtie2 (Li and Durbin 2009, Li and Durbin 2010; Langmead and Salzberg 2012; Kim et al., 2019). The standard output of these tools is in SAM (Sequence Alignment/Map) format, or its binary version, BAM (Binary Alignment/MAP) format. Both formats records the detailed results from read alignment (Li et al., 2009; Li 2011). In the SAM/BAM file, each alignment line has 11 mandatory fields such as mapping position, flag, CIGAR string and mapping quality (Li et al., 2008). Fuseq-WES first filters out reads with mapping quality less than 30, and then extracts the discordant and split reads based on the flag and CIGAR string fields. As shown in Figure 1, the discordant reads are a pair of reads mapping to each side of the fusion gene and the reads are spanning the fusion junction; while the split reads are those overlapping with the fusion junction directly. We keep these two types of reads and exclude the other reads which are mapped to reference perfectly.

### 2.3 Build the Fusion Equivalence Class

**FIGURE 2:**
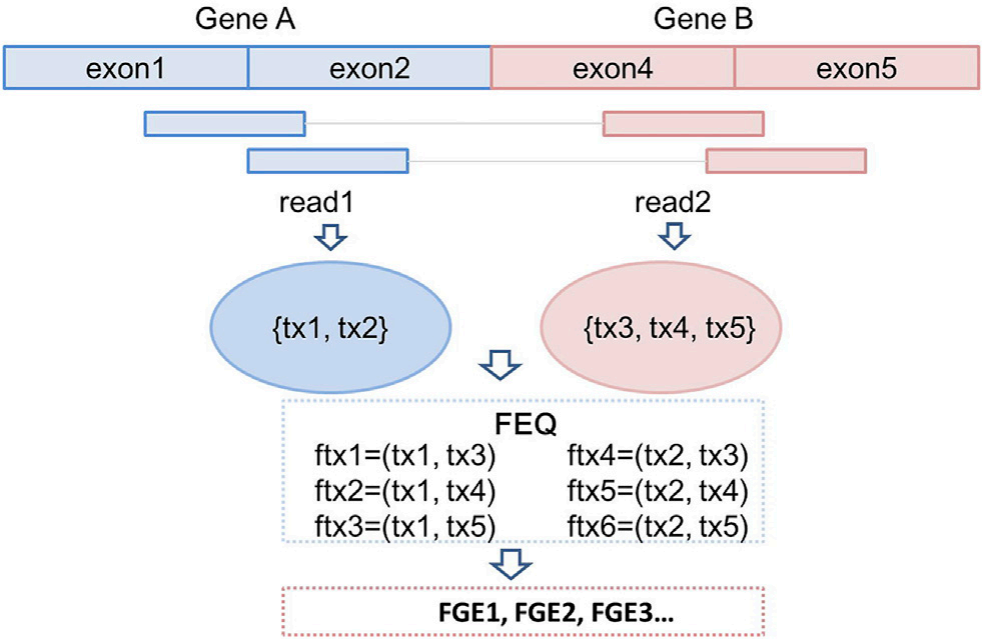
Construction of fusion equivalence class and fusion transcripts; prediction of fusion genes.

All the discordant and split reads are first annotated with the information of mapping position, chromosome, gene names and transcript names. In this step, a GTF (General Transfer Format) file recording the characteristics of genome/transcriptome structure is provided as input (Breese and Liu 2013). In our previous study, we have introduced a novel concept, fusion equivalence class (FEQ), to predict high-confidence fusion gene from discordant reads (Vu et al., 2018). As shown in Figure 2, we assume that there is a pair of discordant reads, read1 and read2, mapping to the constituent parts of fusion gene GeneA-GeneB. The read1 is mapped to transcript tx1 and tx2, and read2 is mapped tx3, tx4 and tx5. We then produce the FEQ, which is the possible combination of fusion transcripts (ftx). In this example, there are six fusion transcripts generated from read1 and read2 (ftx1 to ftx6). Thus, the FEQ describes the reads sharing between potential fusion genes. Based on the FEQ sets and the information of annotation, we infer the corresponding fusion genes (FGEs). In this case, read1 and read2 contribute one read-pair as the supporting reads for the FGEs candidates.

#### 2.3.1 Fusion Gene Candidates From Split Reads

According to the definition, split reads overlap with the fusion breakpoints, thus provide direct evidence supporting a potential fusion event (Haas et al., 2017). For each split read, we set the threshold that the maximum number of genes it can map to as two, which means that a split read map to more than two distinct genes will be discarded. Also, if the split reads are from the same read pair and were overlapping with each other, the non-overlap region should be over 20 bases.

### 2.4 Statistical Tests and Filters

In this step, several statistical tests and filters are applied to exclude the false positives. For example, in terms of the general features, the chromosomes of fusion genes are limited to autosome 1–22 and chromosome X and Y. The constituent genes should be protein-coding genes and the distance between the two genes from the same chromosome must be large enough (10 kilo bases in this study). Also, we set the minimum number of supporting read pairs of a fusion event to be one. Apart from the filtering criteria regarding the general characteristics, another primary factor causing false positives is paralog genes with extremely similar sequences. To address this issue, we implement an RNA-seq simulation to detect paralogs genes/transcripts. The simulation scheme is similar with what we did in our previous study (Deng et al., 2020). We apply an R package Polyester to simulate an artificial sample, in which all the transcripts are assigned a large number of reads to be expressed, e.g., 10,000 (Frazee et al., 2015). After reads mapping, we summarize the reads sharing between different transcripts, which is an important indicator for sequence similarity in transcripts. We then extend the similar sequences from transcript level to gene level, and determine a set of genes that are paralogs. If two genes from a fusion event are paralogs, this fusion gene will be discarded as false positive.

### 2.5 Real whole-Exome Sequencing Datasets From Cancer Cohorts

In this study, we employ our method to several real WES datasets including the BeatAML cohort, TCGA-AML cohort and the Prostate Biomarkers cohort (Network 2013; Nordström et al., 2017; Tyner et al., 2018). All of these studies provide rich resources containing RNA-seq, exome-seq, health record, genetic mutation and biomarker data. We first detect the fusion genes from RNA-seq data and then utilize Fuseq-WES to validate the fusion candidates using whole-exome sequencing data.

#### 2.5.1 BeatAML Cohort

The BeatAML cohort includes a total of 672 tumor specimens representing 562 unique patients, which gives a comprehensive genomic landscape of AML. Specimens from bone marrow and peripheral blood were obtained from patients with informed consent (Tyner et al., 2018). The exome sequencing was performed on 531 patients using the Illumina Nextera RapidCapture Exome capture probes and protocols. After quality control and library preparation, the DNA segments were sequenced using the HiSeq 2500 with paired end 100-cycle protocols. The WES data were then mapped to reference genome using BWA MEM version 0.7.10 (Li 2018). The SAM files were sorted and converted as BAM format using SortSam (do Valle et al., 2016). Also, the BeatAML study has performed RNA sequencing on 411 patients, the samples were processed using Agilent SureSelect Strand-Specific RNA Library Preparation Kit. Reads were sequenced on HiSeq 2500 using a 100-cycle paired-end protocol and saved as FASTQ files (Holm et al., 2019).

#### 2.5.2 TCGA-Acute Myeloid Leukemia Cohort

The TCGA-AML cohort represents another primary project investigating the genomic and epigenomic landscape of AML (Network 2013). In this study, whole-exome sequencing was performed on 150 adult patients using matched tumor and skin tissues. The exome library was constructed and sequenced on either an Illumina HiSeq 2000 or Illumina GAIIX 76-bp platform. The raw reads were aligned to human reference genome using BWA and saved in BAM files. The RNA sequencing was performed on 179 cases using 76-bp pair-end protocols. All datasets were deposited through the Cancer Genome Atlas data portal (https://tcga-data.nci.nih.gov/tcga) for public access.

#### 2.5.3 Prostate Biomarkers Cohort

Prostate cancer is a genetically heterogeneous disease which remains the most common and deadliest malignancy among Swedish men (Nordström et al., 2017). The metastatic castrate-resistant prostate cancer (mCRPC), where the tumor continues to progress regardless of low testosterone level, is one of the most aggressive cancer subtypes (Zhang et al., 2017). The Prostate Biomarkers (ProBio) cohort was built to evaluate the therapeutically predictive markers in mCRPC. A total of 750 men were recruited and randomized to receive either standard of care or treatment with medications. The germline DNA and circulating tumor DNA were extracted from blood samples and then sequenced using the targeted deep-sequencing approach. The TMPRSS2-ERG fusion gene is the most frequent genomic mutation in prostate cancer, which can be found in 55% of cases. From the ProBio group, we obtain a total of 65 patients and the targeted sequencing data, where the TMPRSS2 and ERG are selected for deep sequencing with an average 1500X read depth. To assess the effect of coverage on the accuracy of Fuseq-WES, we perform a subsampling study by taking a random sample of the reads at coverage 7.5x to 150x.

## 3 Results

### 3.1 Fusion Gene Detection in Acute Myeloid Leukemia Patients

We first apply the Fuseq-WES method to validate the fusion genes in BeatAML dataset. PML-RARA is a well characterized fusion gene in acute promyelocytic leukemia (APL), which is a clinically and biologically unique subtype of AML (Liquori et al., 2020). The fusion is produced as the consequence of balanced translocation t (15; 17) (q24; q21), which involves the PML gene on chromosome 15 and the RARA gene on chromosome 17. From the RNA-seq data, we detect 16 samples harboring this chimeric gene. In BeatAML project, we find 11 matched samples with available exome sequencing data. We run the detection pipeline on these samples and validate the PML-RARA in four out of 11 samples. Table 1 shows the number of discordant and split reads supporting the fusion event. For example, in Sample 20–00147, there are five discordant reads and seven split reads mapped to the fusion gene. For Sample 13–00226, 14–00831 and 20–00566 the total number of supportive reads are four, two and two, respectively.

**TABLE 1:** Number of supporting reads for PML-RARA in BeatAML samples.

Sample ID	Fusion	Discordant Read	Split Read	Total
Sample_13-00 226	PML-RARA	2	2	4
Sample_14-00 831	PML-RARA	0	2	2
Sample_20-00 147	PML-RARA	5	7	12
Sample_20-00 566	PML-RARA	1	1	2

The inversion involving p13 and q22 segment on chromosome 16 leads to the fusion of CBFB and MYH11. The CBFB-MYH11 is consistently observed in patients with AML subtype M4Eo (do Valle et al., 2016). Several studies have shown that the chimeric protein product can inhibit differentiation of hematopoietic cells and contribute to the development of AML with the presence of additional alterations. Table 2 shows that in the BeatAML dataset, the fusion event is detected in 25 patients using RNA-seq data. There are 24 cases with matched whole-exome sequencing data; among which, we find that 15 samples are carrying this fusion gene. The validation rate in CBFB-MYH11 (0.63) is much higher than that in PML-RARA (0.36). By checking the gene/exon structure in the UCSC genome browser (Karolchik et al., 2003), we notice that the MYH11 has a total of 43 exons, which gives a high exon density considering that the gene length is 153,896 bp.

**TABLE 2:** The number of samples harboring fusion genes detected from RNA-seq data; number of matched samples with exome sequencing data and number of samples carrying fusion genes identified using WES data in BeatAML and TCGA dataset, respectively.

	BeatAML RNA-seq data	WES data	Fuseq-WES
PML-RARA	16	11	4
CBFB-MYH11	25	24	15
RUNX1-RUNX1T1	9	6	0
—	TCGA RNA-seq data	WES data	Fuseq-WES
PML-RARA	16	6	3
CBFB-MYH11	11	6	0
RUNX1-RUNX1T1	7	4	2

The formation of RUNX1-RUNX1T1 fusion is due to the balanced translocation between chromosome 8 and 21, which can be identified in 5–12% of AML cases (Grinev et al., 2015). In the BeatAML data, we detect nine patients carrying this fusion gene. There are six matched samples with WES data available. However, as Table 2 shows, we are not able to detect the RUNX1-RUNX1T1 fusion in any of the six samples.

We apply the same analysis scheme on the TCGA-AML data to validate the fusion genes. The PML-RARA fusion is detected in 16 samples from RNA-seq data. We obtain six samples with matched exome sequencing data and the fusion event is identified in three of them. For CBFB-MYH11, the RNA-seq data shows that a total of 11 samples carry the fusion and there are six of them having exome sequencing data. Surprisingly, unlike the high validation rate in BeatAML data, none of the six samples having CBFB-MYH11 detected. The RUNX1-RUNX1T1 is identified in seven samples using RNA-seq and is confirmed in two out of four samples with WES data available.

### 3.2 Detection of TMPRSS2-ERG in Prostate Cancer

The Prostate Biomarkers (ProBio) project implements a targeted deep-sequencing approach to characterize the genomic profile of selected genes. TMPRSS2-ERG (TE) is a predominant fusion gene with a 55% prevalence in prostate cancer patients. From the project we obtain 65 samples with targeted sequencing data. We first use four separate tools for fusion gene detection, which include SvABA (Wala et al., 2018), LUMPY (Layer et al., 2014), GRIDSS (Cameron et al., 2017) and an in-house python-based tool named SVcaller. The fusions identified by at least two of the four tools are called positive. All positive-fusion calls for TMPRSS2-ERG are manually verified using the Intergrative Genomics Viewer (IGV); for convenience, we refer to this final call as the IGV call. We then employ the Fuseq-WES pipeline to detect fusion events. Table 3 shows the comparison between the two detection strategies.

**TABLE 3:** Comparison of detection results for TMPRSS2-ERG fusion in ProBio patients using the IGV and Fuseq-WES methods. Overall there is 91% concordance between the two methods.

	Positive IGV	Negative IGV	Total
Postive Fuseq-WES	36	5	41
Negative Fuseq-WES	1	23	24
Total	37	28	65

The IGV call identifies 37 patients to be carrying the TE fusion, while the remaining 28 are TE negative. Using the Fuseq-WES approach, the TE fusion is identified in 41 samples and absent in 24 samples. Overall, the results are concordant in 36 TE positive and 23 TE negative cases, indicating a 91% agreement between the two analyses. The detailed number of mapped and split reads supporting the TE fusion is given in Supplementary Table S1. For the five discordant cases identified as negative by IGV and positive by Fuseq-WES, the number of total supporting reads are 34, 16, 9, 4 and 2. We investigate the reason for discordance: the fusion junctions discovered by Fuseq-WES contain no short insertion that is frequently observed in true fusions (Wala et al., 2018).

We further investigate how sequencing depth impacts on the accuracy of Fuseq-WES. First, we randomly select 10 samples for which the TE fusion is validated manually. Then, for each sample we randomly subsample the reads from original data (1500X coverage) to generate five lower-coverage samples including 150x (10%), 75x (5%), 30x (2%), 15x (1%) and 7.5x (0.05%). Finally, we perform Fuseq-WES for all 10 samples and report in Table 4. The results show that the decrease of sample coverage is strongly correlated with the reduction of validation rate and the number of supporting reads of TE fusion. When the coverage is comparable to the actual WES data of TCGA-AML and BeatAML, the validation rate of TE fusion is close to validation rate of the cohorts: 0.7, 0.5, and 0.4 for 30x, 15x, and 7.5x, respectively. Thus a coverage of at least 75x seems needed to get high accuracy.

**TABLE 4:** Fuseq-WES detection results (in terms of the number of supporting reads) for TMPRSS2-ERG fusion in 10 ProBio samples. For each ProBio sample, we obtained random subsamples of the reads of the original data at various lower-coverage levels.

Sample	150x (10%)	75x (5%)	30x (2%)	15x (1%)	7.5x (0.05%)
1	17	12	6	2	0
2	3	1	0	0	0
3	38	17	9	5	2
4	5	3	0	0	0
5	5	3	1	0	0
6	2	1	2	0	0
7	1	1	0	0	0
8	160	64	28	12	4
9	5	2	2	2	1
10	65	31	16	7	5

## 4 Discussion and Conclusion

In this study, we aim to exploit the potential value of whole exome sequencing data in the context of fusion gene detection. We develop a new method named Fuseq-WES and apply it to several representative cancer datasets. In two of the most comprehensive cohorts of AML, i.e. BeatAML and TCGA-AML, we identify and validate three fusion genes, PML-RARA, CBFB-MYH11 and RUNX1-RUNX1T1, which play important roles in the development and progression of AML. In the Prostate Biomarker dataset, we validate the most recurrent fusion gene, TMPRSS2-ERG, and achieve a 91% concordance using two detection schemes.

Compared with the identification results using RNA-seq data, we find that only a fraction of the fusion genes could be detected in the exome sequencing data. For example, from the BeatAML dataset, we obtain 11 samples with exome-seq data for PML-RARA, and four of them are successfully identified to carry this chimeric alteration. In the TCGA-AML cohort, there are six exome-seq samples and we validate three of them carrying the PML-RARA fusion. High-confidence detection of fusion genes mainly depends on split reads. Thus, we also summarize the number of split reads in the WES and RNA-seq data (Supplementary Table S2). In the BeatAML dataset, the median number of split reads in WES is 15, while in the matched RNA-seq samples the median number is 55. From the TCGA-AML dataset, the median number of split reads in WES data is only two, and the number in RNA-seq samples is nine. These results indicate that WES has three times fewer split-reads than RNA-seq, which partially explains the small number of validated samples using exome-seq data.

An obvious limitation of WES is that only the exome region is targeted from genomic DNA for sequencing. In this case, if a fusion junction is located in an intronic region that is out of the targeted enrichment of exonic regions, the exome sequencing is unable to capture that break point. In contrast, the RNA-seq holds an inherent advantage over exome-seq, by which the non-coding regions are removed to produce mature messenger RNA (mRNA), so that the chimeric junction is detectable by subsequent sequencing. Therefore, a fusion gene can be identified using WES data only if the break point is located in or close to the exonic regions. Another limitation of WES is the short read length, especially the length of valid split reads covering the fusion junction. We have drawn a conceptual figure in Supplementary Figure S1 to illustrate the fusion detection near the exon junction region using WES and RNA-seq reads. As the figure shows, when the breakpoints are close enough to the exon boundary of Gene A and Gene B, the exome-seq reads still have the chance to cover the fusion junction and thus capture the fusion event.

We also find that the validation rate is highly associated with gene-exon structure and the read coverage in WES data. A gene with dense exon distribution and the sequencing data with high coverage could facilitate the fusion detection. For the CBFB-MYH11 fusion, in the BeatAML data we identify a total of 15 samples with the mutation among 24 WES cases. The high validation rate could be due to that the MYH11 has 43 exons with the gene length of ∼154,000 bp, which represents a high density covering the whole region of the gene. However, using the TCGA-AML data, we cannot validate the fusion gene in any of the six cases with WES data available. From the BAM file we notice that the median read-depth of TCGA-AML and BeatAML samples are 15x and 40x, respectively, which is lower than the 75x threshold as indicated in Table 4. Altogether, we conclude that the higher validation rate is directly related to the exon density and read coverage in the exome sequencing data.

This observation is confirmed by the analysis using prostate cancer data. The ProBio project applies a targeted sequencing method including the exons and introns, and provides ultra-deep sequenced data with read-depth up to 1500x. We achieve an overall agreement in 91% of the 65 cancer patients. A subsampling study of the prostate data suggests that at least ∼75x is needed for Fuseq-WES to get high sensitivity and accurate results. We have summarized the coverage of WES data of 23 primary cancers in The Cancer Genome Atlas (TCGA) database, which is presented in Supplementary Table S3. For example, the coverages of glioblastomas and lung adenocarcinoma are about 138x and 98x, respectively (Brennan et al., 2013; Collisson et al., 2014). The high read coverage makes these datasets promising resources to detect fusion genes using Fuseq-WES method. Taken together, we recommend the implementation of Fuseq-WES on high-coverage exome-seq and the targeted sequencing data to detect fusion genes. With the increasing number of exome-seq data generated and published, we anticipate that the WES will yield insightful results of fusion gene detection in cancer studies.

## Data Availability

The original contributions presented in the study are included in the article/Supplementary Material, further inquiries can be directed to the corresponding authors.
