# Supplementary material for: Fusion Gene Detection Using Whole-Exome Sequencing Data in Cancer Patients
Source: Front Genet. 2022 Feb 16;13:820493. doi: 10.3389/fgene.2022.820493 (PMC8888970; doi:10.3389/fgene.2022.820493)
Supplement: Supplementary file 1 [file DataSheet1.docx]

Supplementary document

Fusion gene detection using whole-exome sequencing data in cancer patients

by Wenjiang Deng et al.,

**TABLES**

**Table S1**：The detailed number of mapped and split reads supporting the TMPRSS2-ERG fusion using the Fuseq-WES method in the ProBio samples.

| Sample | Mapped Read | Split Read | Total |
| --- | --- | --- | --- |
| ProBio_36 | 249 | 1241 | 1490 |
| ProBio_28 | 216 | 1270 | 1486 |
| ProBio_26 | 238 | 1035 | 1273 |
| ProBio_51 | 82 | 627 | 709 |
| ProBio_27 | 141 | 488 | 629 |
| ProBio_37 | 174 | 365 | 539 |
| ProBio_34 | 31 | 428 | 459 |
| ProBio_32 | 148 | 294 | 442 |
| ProBio_44 | 161 | 247 | 408 |
| ProBio_23 | 180 | 183 | 363 |
| ProBio_52 | 17 | 318 | 335 |
| ProBio_15 | 70 | 254 | 324 |
| ProBio_43 | 24 | 117 | 141 |
| ProBio_49 | 92 | 40 | 132 |
| ProBio_14 | 55 | 74 | 129 |
| ProBio_13 | 66 | 61 | 127 |
| ProBio_53 | 46 | 67 | 113 |
| ProBio_20 | 36 | 63 | 99 |
| ProBio_25 | 17 | 75 | 92 |
| ProBio_11 | 42 | 45 | 87 |
| ProBio_47 | 32 | 52 | 84 |
| ProBio_45 | 11 | 65 | 76 |
| ProBio_35 | 17 | 53 | 70 |
| ProBio_62 | 70 | 0 | 70 |
| ProBio_12 | 12 | 35 | 47 |
| ProBio_61 | 47 | 0 | 47 |
| ProBio_17 | 10 | 28 | 38 |
| ProBio_1 | 9 | 25 | 34 |
| ProBio_55 | 13 | 18 | 31 |
| ProBio_57 | 13 | 17 | 30 |
| ProBio_48 | 5 | 24 | 29 |
| ProBio_10 | 13 | 15 | 28 |
| ProBio_16 | 8 | 14 | 22 |
| ProBio_39 | 7 | 10 | 17 |
| ProBio_6 | 3 | 13 | 16 |
| ProBio_63 | 13 | 0 | 13 |
| ProBio_2 | 0 | 9 | 9 |
| ProBio_24 | 0 | 9 | 9 |
| ProBio_3 | 4 | 0 | 4 |
| ProBio_4 | 2 | 0 | 2 |
| ProBio_29 | 1 | 0 | 1 |
| ProBio_5 | 0 | 0 | 0 |
| ProBio_7 | 0 | 0 | 0 |
| ProBio_8 | 0 | 0 | 0 |
| ProBio_9 | 0 | 0 | 0 |
| ProBio_18 | 0 | 0 | 0 |
| ProBio_19 | 0 | 0 | 0 |
| ProBio_21 | 0 | 0 | 0 |
| ProBio_22 | 0 | 0 | 0 |
| ProBio_30 | 0 | 0 | 0 |
| ProBio_31 | 0 | 0 | 0 |
| ProBio_33 | 0 | 0 | 0 |
| ProBio_38 | 0 | 0 | 0 |
| ProBio_40 | 0 | 0 | 0 |
| ProBio_41 | 0 | 0 | 0 |
| ProBio_42 | 0 | 0 | 0 |
| ProBio_46 | 0 | 0 | 0 |
| ProBio_50 | 0 | 0 | 0 |
| ProBio_54 | 0 | 0 | 0 |
| ProBio_56 | 0 | 0 | 0 |
| ProBio_58 | 0 | 0 | 0 |
| ProBio_59 | 0 | 0 | 0 |
| ProBio_60 | 0 | 0 | 0 |
| ProBio_64 | 0 | 0 | 0 |
| ProBio_65 | 0 | 0 | 0 |

**Table S2**: The number of supporting reads in WES and RNA-seq data from the ourput of Fuseq-WES

| BeatAML WES data | supportCount | SR | MR | FusionGene |
| --- | --- | --- | --- | --- |
| Sample_13-00226 | 4 | 2 | 2 | PML-RARA |
| Sample_14-00831 | 2 | 2 | 0 | PML-RARA |
| Sample_20-00147 | 12 | 7 | 5 | PML-RARA |
| Sample_20-00566 | 2 | 1 | 1 | PML-RARA |
| Sample_13-00338 | 2 | 2 | 0 | CBFB-MYH11 |
| Sample_13-00487 | 16 | 16 | 0 | CBFB-MYH11 |
| Sample_14-00359 | 6 | 5 | 1 | CBFB-MYH11 |
| Sample_15-00045 | 25 | 25 | 0 | CBFB-MYH11 |
| Sample_15-00331 | 10 | 10 | 0 | CBFB-MYH11 |
| Sample_15-00702 | 107 | 107 | 0 | CBFB-MYH11 |
| Sample_15-00819 | 9 | 9 | 0 | CBFB-MYH11 |
| Sample_15-00850 | 97 | 39 | 58 | CBFB-MYH11 |
| Sample_16-00001 | 2 | 1 | 1 | CBFB-MYH11 |
| Sample_20-00116 | 6 | 6 | 0 | CBFB-MYH11 |
| Sample_20-00126 | 5 | 3 | 2 | CBFB-MYH11 |
| Sample_20-00153 | 34 | 33 | 1 | CBFB-MYH11 |
| Sample_20-00172 | 2 | 2 | 0 | CBFB-MYH11 |
| Sample_20-00174 | 10 | 8 | 2 | CBFB-MYH11 |
| Sample_20-00542 | 5 | 5 | 0 | CBFB-MYH11 |
| RNA-seq data | supportCount | SR | MR | FusionGene |
| 13-00204 | 41 | 32 | 9 | PML--RARA |
| 13-00226 | 41 | 27 | 0 | PML--RARA |
| 14-00425 | 44 | 32 | 12 | RARA--PML |
| 14-00425 | 41 | 31 | 10 | PML--RARA |
| 14-00831 | 14 | 10 | 4 | PML--RARA |
| 14-00831 | 10 | 8 | 2 | RARA--PML |
| 15-00338 | 98 | 45 | 0 | RARA--PML |
| 15-00338 | 61 | 48 | 13 | PML--RARA |
| 15-00837 | 461 | 347 | 114 | CBFB--MYH11 |
| 13-00338 | 20 | 11 | 9 | CBFB--MYH11 |
| 14-00279 | 100 | 59 | 41 | CBFB--MYH11 |
| 14-00359 | 66 | 46 | 20 | CBFB--MYH11 |
| 14-00423 | 54 | 30 | 24 | CBFB--MYH11 |
| 13-00496 | 21 | 15 | 6 | CBFB--MYH11 |
| 14-00798 | 44 | 30 | 14 | CBFB--MYH11 |
| 13-00487 | 57 | 40 | 17 | CBFB--MYH11 |
| 14-00613 | 34 | 24 | 10 | CBFB--MYH11 |
| 15-00045 | 37 | 25 | 12 | CBFB--MYH11 |
| 15-00331 | 38 | 17 | 21 | CBFB--MYH11 |
| 16-00001 | 105 | 67 | 38 | CBFB--MYH11 |
| 15-00702 | 120 | 70 | 50 | CBFB--MYH11 |
| 15-00819 | 116 | 69 | 47 | CBFB--MYH11 |
| 15-00837 | 461 | 347 | 114 | CBFB--MYH11 |
| 15-00850 | 121 | 68 | 53 | CBFB--MYH11 |
| 14-00643 | 52 | 21 | 0 | RUNX1--RUNX1T1 |
| 13-00615 | 88 | 40 | 0 | RUNX1--RUNX1T1 |
| 13-00245 | 43 | 16 | 27 | RUNX1--RUNX1T1 |
| 15-00756 | 253 | 22 | 0 | RUNX1--RUNX1T1 |
| 15-00763 | 412 | 39 | 373 | RUNX1--RUNX1T1 |
| 15-00807 | 119 | 10 | 109 | RUNX1--RUNX1T1 |
| TCGA AML WES | supportCount | SR | MR | FusionGene |
| TCGA-AB-2803-03B-01W-0728-08 | 2 | 0 | 2 | PML-RARA |
| TCGA-AB-2841-03B-01W-0728-08 | 3 | 2 | 1 | PML-RARA |
| TCGA-AB-2872-03A-01W-0732-08 | 1 | 1 | 0 | PML-RARA |
| TCGA-AB-2897-03A-01W-0733-08 | 3 | 2 | 1 | PML-RARA |
| TCGA-AB-2819-03B-01W-0728-08 | 7 | 2 | 5 | RUNX1--RUNX1T1 |
| TCGA-AB-2886-03A-01W-0732-08 | 1 | 1 | 0 | RUNX1--RUNX1T1 |
| TCGA AML RNA-seq | supportCount | SR | MR | FusionGene |
| TCGA-AB-2840 | 60 | 3 | 57 | RARA--PML |
| TCGA-AB-2840 | 52 | 1 | 51 | PML--RARA |
| TCGA-AB-2803 | 29 | 1 | 28 | PML--RARA |
| TCGA-AB-2897 | 47 | 3 | 44 | PML--RARA |
| TCGA-AB-2897 | 10 | 0 | 8 | RARA--PML |
| TCGA-AB-2862 | 103 | 6 | 97 | PML--RARA |
| TCGA-AB-2841 | 63 | 8 | 0 | RARA--PML |
| TCGA-AB-2841 | 39 | 1 | 38 | PML--RARA |
| TCGA-AB-2872 | 31 | 2 | 29 | PML--RARA |
| TCGA-AB-2872 | 8 | 0 | 8 | RARA--PML |
| TCGA-AB-2886 | 656 | 48 | 608 | RUNX1--RUNX1T1 |
| TCGA-AB-2875 | 465 | 41 | 424 | RUNX1--RUNX1T1 |
| TCGA-AB-2819 | 259 | 11 | 248 | RUNX1--RUNX1T1 |
| TCGA-AB-2937 | 354 | 28 | 326 | RUNX1--RUNX1T1 |
| TCGA-AB-2937 | 10 | 6 | 0 | RUNX1T1--RUNX1 |
| TCGA-AB-2815 | 55 | 10 | 45 | CBFB--MYH11 |
| TCGA-AB-2815 | 4 | 0 | 4 | MYH11--CBFB |
| TCGA-AB-2914 | 57 | 0 | 50 | CBFB--MYH11 |
| TCGA-AB-2828 | 84 | 9 | 75 | CBFB--MYH11 |
| TCGA-AB-2846 | 50 | 10 | 40 | CBFB--MYH11 |
| TCGA-AB-2888 | 21 | 3 | 18 | CBFB--MYH11 |
| TCGA-AB-2881 | 51 | 11 | 40 | CBFB--MYH11 |

**Table S3**: The coverage of WES data of 23 primary cancers in The Cancer Genome Atlas (TCGA) database.

| TCGA code | Sequencing depth  (average) | | Publication |  |  |
| --- | --- | --- | --- | --- | --- |
| ACC | 20 | https://www.cell.com/cancer-cell/fulltext/S1535-6108(16)30160-X | | | |
| BCLA | 30 | https://www.nature.com/articles/nature12965 | | | |
| BRCA | 20 | <https://www.nature.com/articles/nature11412> | | | |
| CESC | 49 | https://www.nature.com/articles/nature21386 | | | |
| COAD | 20 | <https://www.nature.com/articles/nature11252> | | | |
| ESCA | 20 | https://www.nature.com/articles/nature20805 | | | |
| GBM | 138 | <https://www.cell.com/cell/fulltext/S0092-8674(13)01208-7> | | | |
| HNSC | 30 | https://www.nature.com/articles/nature14129 | | | |
| KIRC | 20 | https://www.nature.com/articles/nature12222 | | | |
| KIRP | 20 | https://www.nejm.org/doi/full/10.1056/NEJMoa1505917 | | | |
| LGG | 30 | <https://www.nejm.org/doi/full/10.1056/NEJMoa1402121> | | | |
| LIHC | 100 | <https://www.cell.com/cell/fulltext/S0092-8674(17)30639-6> | | | |
| LUAD | 97.6 | https://www.nature.com/articles/nature13385 | | | |
| LUSC | 30 | <https://www.nature.com/articles/nature11404> | | | |
| PAAD | 405 | https://www.cell.com/cancer-cell/fulltext/S1535-6108(17)30299-4 | | | |
| PCPG | 150 | https://www.cell.com/cancer-cell/fulltext/S1535-6108(17)30001-6 | | | |
| PRAD | 100 | https://www.cell.com/cell/fulltext/S0092-8674(15)01339-2 | | | |
| SARC | 20 | https://www.cell.com/cell/fulltext/S0092-8674(17)31203-5 | | | |
| SKCM | 30 | <https://www.cell.com/cell/fulltext/S0092-8674(15)00634-0> | | | |
| STAD | 30 | https://www.nature.com/articles/nature13480 | | | |
| THCA | 97 | https://www.cell.com/cell/fulltext/S0092-8674(14)01238-0 | | | |
| UCEC | 20 | https://www.nature.com/articles/nature12113 | | | |

**FIGURES**


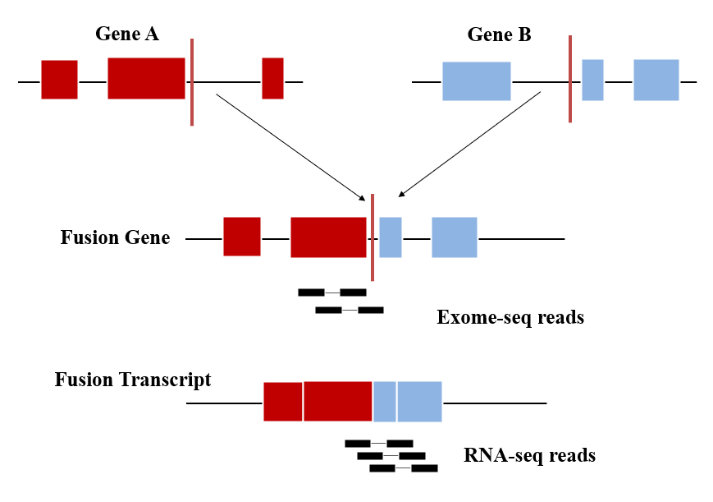


**Figure S1**: the fusion detection near the exon junction region using WES and RNA-seq reads. When the breakpoints in Gene A and Gene B are close to the exon boundary, the exome-seq reads still have the possibility to cover the fusion junction and thus capture the fusion event.
